# Two case series reports: 8 cases of arachnoid Temporoparietal cysts (middle fossa & sylvian fissure) and 2 cases of chronic subdural hematoma

**Published:** 2012-11

**Authors:** Ali Meshkini, Mohammad Meshkini

**Affiliations:** ^*a*^Department of Medical Faculty, Tabriz University of Medical Sciences, Tabriz, Iran.

## Abstract

**Background:**

Arachnoid cysts are common intracranial space-occupying lesions which are often found in middle fossa and temporal regions of the skull. Many of these lesions are asymptomatic but some might appear as space-occupying lesions. Almost arachnoid cyst rupture, either following a trauma or spontaneously can result in intracystic hemorrhage, subdural hematoma and hygroma. The present study presents two case series including 8 cases of arachnoid cysts in temporal region and 2 cases of subdural hemorrhage.

**Methods:**

Demographic data and clinical and neuroimaging features of 8 patients were evaluated.

**Results:**

A total of 8 patients with arachnoid cysts in temporal region were assessed: age range 3 to 27 years old, 5 male and 3 female. The most important complains of the patients during their visit were seizure (3 cases), headache (4 cases), increased head circumference (1 case), parietotemporal arachnoid cyst in right (4 cases) and left hemisphere (4 cases). The conservative treatment and follow-up were performed in 6 out of 8 patients. In the other 2 patients, for craniotomy surgery with hematoma evacuation was performed. Furthermore, in the surgery the fenestration of arachnoid cyst wall into the basal cisterns as well as low pressure cysto-peritoneal shunt was performed.

**Conclusions:**

The risk of annual hemorrhage for patients with arachnoid cyst is very low. However, when the hemorrhage occurs it is treated by hematoma evacuation in most cases, but sometimes there is a need for fenestration of the cyst into basal cisterns under endoscopy, microsurgical or cystoperitoneal shunt.

**Keywords:**

Arachnoid cyst, Middle fossa, Chronic subdural hematoma


					Volume 4, Suppl. 1 Nov 2012
Publisher: Kermanshah University of Medical Sciences
URL: http://www.jivresearch.org
Copyright:
Congress of Iranian Neurosurgeons, 14-16 November 2012, Kermanshah, Iran
Paper No. 15
Two case series reports: 8 cases of arachnoid Temporoparietal
cysts (middle fossa & sylvian fissure) and 2 cases of chronic
subdural hematoma
Ali Meshkini a,*
, Mohammad Meshkini a
a
Department of Medical Faculty, Tabriz University of Medical Sciences, Tabriz, Iran.
Abstract:
Background: Arachnoid cysts are common intracranial space-occupying lesions which are often found in middle fossa
and temporal regions of the skull. Many of these lesions are asymptomatic but some might appear as space-
occupying lesions. Almost arachnoid cyst rupture, either following a trauma or spontaneously can result in intracystic
hemorrhage, subdural hematoma and hygroma. The present study presents two case series including 8 cases of
arachnoid cysts in temporal region and 2 cases of subdural hemorrhage.
Methods: Demographic data and clinical and neuroimaging features of 8 patients were evaluated.
Results: A total of 8 patients with arachnoid cysts in temporal region were assessed: age range 3 to 27 years old, 5
male and 3 female. The most important complains of the patients during their visit were seizure (3 cases), headache
(4 cases), increased head circumference (1 case), parietotemporal arachnoid cyst in right (4 cases) and left
hemisphere (4 cases). The conservative treatment and follow-up were performed in 6 out of 8 patients. In the other 2
patients, for craniotomy surgery with hematoma evacuation was performed. Furthermore, in the surgery the
fenestration of arachnoid cyst wall into the basal cisterns as well as low pressure cysto-peritoneal shunt was
performed.
Conclusions: The risk of annual hemorrhage for patients with arachnoid cyst is very low. However, when the
hemorrhage occurs it is treated by hematoma evacuation in most cases, but sometimes there is a need for
fenestration of the cyst into basal cisterns under endoscopy, microsurgical or cystoperitoneal shunt.
Keywords:
Arachnoid cyst, Middle fossa, Chronic subdural hematoma
* Corresponding Author at:
Ali Meshkini: Department of Medical Faculty, Tabriz University of Medical Sciences, Tabriz, Iran, Tel: 09141144484,
Email: meshkinia@yahoo.com, (Meshkini A.).

				

